# Willingness of medical students to be examined in a physical examination course

**DOI:** 10.1186/s12909-018-1353-5

**Published:** 2018-10-29

**Authors:** Manuel Burggraf, Julia Kristin, Alexander Wegner, Sascha Beck, Stephanie Herbstreit, Marcel Dudda, Marcus Jäger, Max Daniel Kauther

**Affiliations:** 1 Department of Orthopaedics and Trauma Surgery, University Hospital Essen, University of Duisburg-Essen, Hufelandstr. 55, 45147 Essen, Germany; 2 Department of Otorhinolaryngology, University Hospital Duesseldorf, Heinrich Heine University Duesseldorf, Duesseldorf, Germany

**Keywords:** Physical examination, Clinical skills, Teaching, Medical students, Peer, Medical education

## Abstract

**Background:**

Physical examination courses are an essential part of the education of medical students. The aim of this study was to ascertain the factors influencing students’ motivation and willingness to participate in a physical examination course.

**Methods:**

Students were asked to complete a questionnaire subdivided into five domains: anthropometric data, religiousness, motivation to take part in physical examination courses, willingness to be physically examined at 11 different body regions by peers or a professional tutor and a field for free text.

**Results:**

The questionnaire was completed by 142 medical students. The importance of the examination course was rated 8.7 / 10 points, the score for students’ motivation was 7.8 / 10 points. Willingness to be physically examined ranged from 6 to 100% depending on body part and examiner. Female students were significantly less willing to be examined at sensitive body parts (breast, upper body, groin and the hip joint; *p* = .003 to < .001), depending on group composition and / or examiner. Strictly religious students showed significantly less willingness to undergo examination of any part of the body except the hand (*p* = .02 to < .001). Considering BMI, willingness to be examined showed comparable rates for normal weight and under- / overweight students in general (80% vs. 77%). Concerning the composition of the group for physical examination skills courses, students preferred self-assembled over mixed gender and same gender groups.

**Conclusions:**

Peer physical examination is a method to improve students’ skills. While motivation to participate in and acceptance of the physical examination course appears to be high, willingness to be examined is low for certain parts of the body, e.g. breast and groin, depending on religiousness, gender and examiner. Examination by a professional medical tutor did not lead to higher acceptance. Most students would prefer to choose their team for physical examination courses themselves rather than be assigned to a group.

## Background

Despite technical and medical advances and E-health, the basic physical examination of patients is still a mandatory competence for every physician. Acquired physical examination skills are known to be of lasting value. Data suggests that even greater emphasis should be placed on basic clinical skills [1, 2]. In recent years, there has been a trend in medical curricula away from classical teacher-centred lectures towards clinical skills courses including peer physical examinations by students as well as bedside teaching. All this attention has encouraged many medical schools to revise their curricula and emphasize the importance of teaching more clinical skills and to establish clinical skill centres to provide appropriate training [3–5]. Consequently, objective structured clinical examinations (OSCEs) and mini-clinical evaluation exercises (mini-CEXs) are additionally used as assessment methods [6, 7] to grade the clinical skills of students.

At some teaching hospitals standardised patients are used in medical education to expose students to clinical contexts and facilitate transition to clinical practice. The main limiting factors are costs and availability, not only in countries with public universities in which medical training is free of charge. Hence, the physical examination of real patients without prior teaching can be very challenging for both students and patients [8]. The top three most relevant aspects of pre-clinical preparation for clinical practice are skills training, clinical tutorials with real patients and standardised patients contacts [8]. Mounsey et al. showed that standardised patient role-plays are similar in effectiveness to student role-plays when teaching basic motivational interviewing skills [9].

Peer physical examination is as an experiential method of learning clinical skills where medical students are models for one another [10]. It stands for a teaching method in a supervised environment, including a constructive feedback from tutors, that allows to develop compassion and empathy, to improve communication skills and to gain insight the patient’s psychosocial experiences from being examined [11]. Mentioned negative aspects for students are the perceived pressure to act as model (male > female), the missing possibility for the model to participate in examining and the lack of abnormal signs on students [11]. Student’s gender, the examination of specific, sensitive body regions, religion and ethnicity appear to be potential barriers to some students willingness to participate in PPE [12]. Therefore, the principle of non-maleficence, physically and emotionally through embarrassment or harassment, must be maintained. It is suggested to provide written protocols available to students before classes, information sheets stating the risks and benefits, and to obtain written informed consent from students [10, 13, 14].

Evaluation of student participants is an important resource for implementation and continuous improvement of training programs. The aim of this study was to analyse the motivation and willingness of students to be examined for teaching purposes and to identify potential problem areas in this context. The results might then provide a basis for improvement of the physical examination courses of our and other institutions.

## Methods

Ethical considerations: The study was approved by the local ethics committee of the University Hospital Essen (study number: 11–4891-BO).

Participants and setting: Third-year medical students (first year in clinical training) of the Faculty of Medicine of the University of Duisburg-Essen at the University Hospital of Essen (Germany), who were about to attend the mandatory peer examination course, were queried. The interdisciplinary course, carried out through various clinical departments, is conducted in small groups and supervised by a professional tutor. Following the mandatory introductory lecture, students were asked to complete a questionnaire about peer examination courses.

All of them were of legal age, participated on a voluntary basis and provided written informed consent. They completed a written questionnaire about peer physical examinations in clinical skills courses. Questionnaires lacking information on gender or students who refused participation were excluded. All data was collected in anonymised form.

Questionnaire: We designed a self-administered questionnaire, subdivided into five domains, to document anthropometric and personal data comprising age, gender, height, weight and religious affiliation. Using a 10-point Likert-scale, respondents indicated the importance for them and their motivation in participating (1 = not at all, to 10 = very important and highest motivation, respectively) in the physical examination skills course. Furthermore, the frequency of voluntary participation in physical examination by a tutor in front of a group of students was also recorded. The core element of the questionnaire was the potential willingness to be examined on 11 different body parts by a same gender student, a different gender student, a same gender professional tutor or a different gender professional tutor. The body regions comprised head and neck, hand, arm and shoulder, upper body, breast, abdomen, back, groin, lower leg and foot, knee and the hip joint. Additionally, students were asked for their preferred composition of the group: self-assembled, mixed gender or same gender. Finally, students could write a free text suggestion as to how physical examination skills could be improved.

Groups of students were formed according to gender, body mass index (BMI) and religiousness. Normal weight was defined as a BMI of 18.5 to 25, underweight as a BMI of < 18.5 and overweight as a BMI > 25, respectively.

Pearson’s chi-squared test (χ^2^) was used to analyse the influence of gender, BMI and religiousness. A *p*-value < .05 (2-sided) was considered statistically significant. Additionally, the mean square contingency coefficient (phi coefficient, φ) was computed to give the strength of association. The statistical analysis was performed using IBM® SPSS Statistics 25.

## Results

All (145/145) recruited students filled in the questionnaire. The final study included 142 of 145 completed questionnaires. Three questionnaires were excluded due to missing information regarding gender (Table 1).

**Table 1:** Anthropometric data

	Male students	Female students
Participants (*n* = 142)	*n* = 60 (42%)	*n* = 82 (58%)
Age: 19–52 years	24 (mean) / 23 (median)	23 (mean) / 22 (median)
Height	1.82 m	1.69 m
Weight	79.9 kg	60.8 kg
BMI (both gender:22.5)	24.1	21.2
Normal BMI	*n* = 105 (male + female)	
BMI < 18.5 or > 25	*n* = 30 (male + female)	
Religion	49 Christian	65 Christian
	1 Muslim	8 Muslim
	2 other	4 other
	7 no religious affiliation	4 no religious affiliation
Strength of religiousness	3 strong belief	9 strong belief
	47 less religious	51 less religious

Some respondents did not comment on certain subdomains: age and height: 1 *m*, weight: 1 *m* and 6 *f*, religious affiliation: 1 *m* and 1 *f*, degree of religiousness: 10 *m* and 22 *f*.

The importance of the physical examination course was rated 8.7 out of 10 points (*m* = 8.6; *f* = 8.7), the score for students’ motivation was 7.8 points (*m* = 7.6; *f* = 7.9). Fifteen of sixty males (25%) and 26 of 82 females (32%) never made themselves available for physical examination by a medical tutor, 41 males (68%) and 46 females (56%) up to five times. Four males (7%) and 9 females (11%) were examined more than five times by a medical tutor.

In general, willingness to be physically examined by peers is higher in male than in female students (86% vs. 74%; see Table 2).

**Table 2:** Reported willingness of students to be examined depending on examiner and gender

	Male students	Female students
Overall	86%	74%
Student of same gender	90%	84%
Student of different gender	87%	70%
Tutor of same gender	83%	76%
Tutor of different gender	83%	66%

Nevertheless, both genders preferred to be examined by a peer of the same gender (males 90%; females 86%), whereas willingness to be physically examined by a tutor of the opposite gender was least acceptable (males 83%; females 66%). In fact, the reported willingness strongly depends on the body region to be examined (see Table 3).

**Table 3:** Reported willingness to be examined on different body regions according to group composition and gender

Gender	Same gender student		Different gender student		Same gender tutor		Different gender tutor	
	Male	Female	Male	Female	Male	Female	Male	Female
Head and neck	98%	98%	97%	93%	93%	95%	92%	90%
Hand	98%	100%	98%	99%	93%	99%	92%	96%
Arm and shoulder	97%	96%	95%	93%	93%	93%	90%	89%
Upper body	90%	81%	85%	60%	80%	68%	80%	46%
Breast	88%	39%	82%	7%	75%	24%	75%	6%
Abdomen	92%	88%	85%	77%	80%	83%	80%	72%
Back	92%	89%	85%	81%	83%	84%	82%	76%
Groin	62%	61%	55%	29%	57%	43%	57%	23%
Lower leg and foot	92%	93%	90%	85%	87%	84%	88%	81%
Knee	97%	96%	95%	90%	93%	94%	93%	87%
Hip joint	85%	79%	87%	61%	82%	71%	83%	60%

Willingness to be examined ranged from 6% (breast examination of females by a different gender tutor) to 100% (examination of the hand of females by a same gender student). Examination of the head and neck as well as the hand was consistently accepted by > 90% of the students, irrespective of the examiner. One male student stated no potential willingness to be physically examined at all, irrespective of body region and examiner.

Statistical analysis showed that female students are significantly less willing to undergo breast examination (*p* < .001; φ 0.50 to 0.75), which is irrespective of the examiner (see Table 4).

**Table 4:** Differences in willingness to be examined between male and female students

Gender	Same gender student	Different gender student	Same gender tutor	Different gender tutor
Head and neck	χ^2^ [1] = 0.10 *p* = .75 φ = 0.03	χ^2^ [1] = 1.03 *p* = .31 φ = 0.09	χ^2^ [1] = 0.21 *p* = .65 φ = 0.04	χ^2^ [1] = 0.08 *p* = .77 φ = 0.02
Hand	χ^2^ [1] = 1.38 *p* = .24 φ = 0.10	χ^2^ [1] = 0.05 *p* = .82 φ = 0.02	χ^2^ [1] = 3.03 *p* = .08 φ = 0.15	χ^2^ [1] = 1.42 *p* = .23, φ = 0.10
Arm and shoulder	χ^2^ [1] = 0.01 *p* = .92 φ = 0.01	χ^2^ [1] = 0.31 *p* = .58 φ = 0.05	χ^2^ [1] = 0.02 *p* = .88 φ = 0.01	χ^2^ [1] = 0.04 *p* = .85 φ = 0.02
Upper body	χ^2^ [1] = 2.39 *p* = .12 φ = 0.13	**χ**^**2(1)**^ **= 10.60** ***p*** **= .001** **φ = 0.27**	χ^2^ [1] = 2.42 *p* = .12 φ = 0.13	**χ**^**2(1)**^ **= 16.44** ***p*** **< .001** **φ = 0.34**
Breast	**χ**^**2(1)**^ **= 35.06** ***p*** **< .001** **φ = 0.50**	**χ**^**2(1)**^ **= 80.71** ***p*** **< .001** **φ = 0.75**	**χ**^**2(1)**^ **= 35.75** ***p*** **< .001** **φ = 0.50**	**χ**^**2(1)**^ **= 72.11** ***p*** **< .001** **φ = 0.71**
Abdomen	χ^2^ [1] = 0.55 *p* = .46 φ = 0.06	χ^2^ [1] = 1.46 *p* = .23 φ = 0.10	χ^2^ [1] = 0.20 *p* = .66 φ = 0.04	χ^2^ [1] = 1.21 *p* = .27 φ = 0.09
Back	χ^2^ [1] = 0.27 *p* = .60 φ = 0.04	χ^2^ [1] = 0.49 *p* = .49 φ = 0.06	χ^2^ [1] = 0.02 *p* = .90 φ = 0.01	χ^2^ [1] = 0.75 *p* = .39 φ = 0.07
Groin	χ^2^ [1] = 0.01 *p* = .93 φ = 0.01	**χ**^**2(1)**^ **= 9.55** ***p*** **= .002** **φ = 0.26**	χ^2^ [1] = 2.71 *p* = .10 φ = 0.14	**χ**^**2(1)**^ **= 16.62** ***p*** **< .001** **φ = 0.34**
Lower leg and foot	χ^2^ [1] = 0.05 *p* = .82 φ = 0.02	χ^2^ [1] = 0.67 *p* = .41 φ = 0.07	χ^2^ [1] = 0.06 *p* = .80 φ = 0.02	χ^2^ [1] = 1.57 *p* = .21 φ = 0.11
Knee	χ^2^ [1] = 0.01 *p* = .92 φ = 0.01	χ^2^ [1] = 1.10 *p* = .30 φ = 0.09	χ^2^ [1] = 0.02 *p* = .89 φ = 0.01	χ^2^ [1] = 1.24 *p* = .27 φ = 0.09
Hip joint	χ^2^ [1] = 0.76 *p* = .38 φ = 0.07	**χ**^**2(1)**^ **= 11.30** ***p*** **= .001** **φ = 0.28**	χ^2^ [1] = 2.23 *p* = .14 φ = 0.13	**χ**^**2(1)**^ **= 9.12** ***p*** **= .003** **φ = 0.25**

Pearson’s chi-squared test (χ2) and 2-sided *p*-value together with mean square contingency coefficient (phi coefficient, φ). Significant differences are highlighted in bold (2-sided *p*-value < .05)

Furthermore, in contrast to male students, female students are significantly less willing to be examined by a student or a tutor of the opposite gender at the upper body, the groin and the hip joint (*p* = .003 to < .001; φ 0.25 to 0.34). In fact, males also stated the least willingness (55% to 62%) to be examined on the groin under any constellation.

Considering BMI, willingness to be examined showed comparable rates for normal weight and under- / overweight students in general (80% vs. 77%) and irrespective of examiner (see Table 5).

**Table 5:** Willingness to be examined depending on examiner and body mass index

	Normal weight	Under−/overweight
Overall	80%	77%
Student of same gender	88%	85%
Student of different gender	79%	74%
Tutor of same gender	80%	78%
Tutor of different gender	74%	72%

A statistically significantly higher willingness of normal weight students was only found for the examination of the arm and shoulder region by a same gender student (*p* = .04; φ 0.18) (see Table 6).

**Table 6:** Differences between normal and under−/overweight students in willingness to be examined

BMI	Same gender student	Different gender student	Same gender tutor	Different gender tutor
Head and neck	χ^2^ [1] = 0.21 *p* = .65 φ = 0.04	χ^2^ [1] = 0.48 *p* = .49 φ = 0.06	χ^2^ [1] = 0.16 *p* = .69 φ = 0.04	χ^2^ [1] = 0.12 *p* = .73 φ = 0.03
Hand	χ^2^ [1] = 3.49 *p* = .06 φ = 0.16	χ^2^ [1] = 0.89 *p* = .35 φ = 0.08	χ^2^ [1] = 0.93 *p* = .34 φ = 0.08	χ^2^ [1] = 0.16 *p* = .69 φ = 0.04
Arm and Shoulder	**χ**^**2(1)**^ **= 4.23** ***p*** **= .04** **φ = 0.18**	χ^2^ [1] = 0.67 *p* = .42 φ = 0.07	χ^2^ [1] = 1.93 *p* = .17 φ = 0.12	χ^2^ [1] = 1.59 *p* = .21 φ = 0.11
Upper body	χ^2^ [1] = 1.07 *p* = .30 φ = 0.09	χ^2^ [1] = 0.86 *p* = .35 φ = 0.08	χ^2^ [1] = 1.04 *p* = .31 φ = 0.09	χ^2^ [1] = 0.33 *p* = .56 φ = 0.05
Breast	χ^2^ [1] = 0.26 *p* = .61 φ = 0.04	χ^2^ [1] = 0.00 *p* = .97 φ = 0.00	χ^2^ [1] = 1.45 *p* = .23 φ = 0.10	χ^2^ [1] = 0.76 *p* = .38 φ = 0.08
Abdomen	χ^2^ [1] = 0.41 *p* = .52 φ = 0.06	χ^2^ [1] = 2.02 *p* = .16 φ = 0.12	χ^2^ [1] = 0.12 *p* = .74 φ = 0.03	χ^2^ [1] = 1.03 *p* = .31 φ = 0.09
Back	χ^2^ [1] = 0.05 *p* = .82 φ = 0.02	χ^2^ [1] = 1.04 *p* = .31 φ = 0.09	χ^2^ [1] = 0.55 *p* = .46 φ = 0.06	χ^2^ [1] = 0.58 *p* = .49 φ = 0.07
Groin	χ^2^ [1] = 0.26 *p* = .61 φ = 0.04	χ^2^ [1] = 0.55 *p* = .46 φ = 0.06	χ^2^ [1] = 0.17 *p* = .68 φ = 0.04	χ^2^ [1] = 0.02 *p* = .88 φ = 0.01
Lower leg and foot	χ^2^ [1] = 0.67 *p* = .42 φ = 0.07	χ^2^ [1] = 0.07 *p* = .79 φ = 0.02	χ^2^ [1] = 0.33 *p* = .57 φ = 0.05	χ^2^ [1] = 0.55 *p* = .46 φ = 0.06
Knee	χ^2^ [1] = 0.93 *p* = .34 φ = 0.08	χ^2^ [1] = 0.17 *p* = .69 φ = 0.04	χ^2^ [1] = 0.67 *p* = .42 φ = 0.07	χ^2^ [1] = 0.34 *p* = .56 φ = 0.05
Hip joint	χ^2^ [1] = 1.04 *p* = .31 φ = 0.09	χ^2^ [1] = 0.63 *p* = .43 φ = 0.07	χ^2^ [1] = 0.80 *p* = .37 φ = 0.08	χ^2^ [1] = 0.34 *p* = .56 φ = 0.05

1 Pearson’s chi-squared test (χ2) and 2-sided *p*-value together with mean square contingency coefficient (phi coefficient, φ). Significant differences are highlighted in bold (2-sided *p*-value < .05)

Regarding the influence of religiousness, 12 students stated that they were strictly religious (Muslims, Christians and others). The 12 students with strong religious beliefs showed an overall willingness of 52% to be examined. Compared to students who reported a minor degree of religiousness, the strictly religious students showed significantly less willingness to undergo examination of any part of the body except the hand (see Table 7).

**Table 7:** Differences between strictly religious and less religious students in willingness to be examined

Religiousness	Same gender student	Different gender student	Same gender tutor	Different gender tutor
Head and neck	χ^2^ [1] = 0.12 *p* = .73 φ = 0.03	**χ**^**2(1)**^ **= 12.99** ***p*** **< .001** **φ = 0.34**	**χ**^**2(1)**^ **= 6.53** ***p*** **= .01** **φ = 0.24**	**χ**^**2(1)**^ **= 6.28** ***p*** **= .01** **φ = 0.24**
Hand	χ^2^ [1] = 0.12 *p* = .73 φ = 0.03	χ^2^ [1] = 0.25 *p* = .62 φ = 0.05	χ^2^ [1] = 1.60 *p* = .21 φ = 0.12	χ^2^ [1] = 0.45 *p* = .51 φ = 0.06
Arm and shoulder	χ^2^ [1] = 0.25 *p* = .62 φ = 0.05	**χ**^**2(1)**^ **= 20.30** ***p*** **< .001** **φ = 0.43**	**χ**^**2(1)**^ **= 9.98** ***p*** **< .01** **φ = 0.30**	**χ**^**2(1)**^ **= 13.57** ***p*** **< .001** **φ = 0.35**
Upper body	**χ**^**2(1)**^ **= 11.52** ***p*** **< .01** **φ = 0.32**	**χ**^**2(1)**^ **= 14.59** ***p*** **< .001** **φ = 0.36**	**χ**^**2(1)**^ **= 8.30** ***p*** **< .01** **φ = 0.26**	**χ**^**2(1)**^ **= 6.88** ***p*** **= .01** **φ = 0.25**
Breast	**χ**^**2(1)**^ **= 6.88** ***p*** **= .01** **φ = 0.25**	χ^2^ [1] = 3.06 *p* = .08 φ = 0.17	χ^2^ [1] = 2.47 *p* = .12 φ = 0.15	χ^2^ [1] = 2.26 *p* = .13 φ = 0.14
Abdomen	**χ**^**2(1)**^ **= 17.30** ***p*** **< .001** **φ = 0.40**	**χ**^**2(1)**^ **= 21.29** ***p*** **< .001** **φ = 0.44**	**χ**^**2(1)**^ **= 9.17** ***p*** **< .01** **φ = 0.29**	**χ**^**2(1)**^ **= 12.90** ***p*** **< .001** **φ = 0.34**
Back	**χ**^**2(1)**^ **= 11.34** ***p*** **< .01** **φ = 0.32**	**χ**^**2(1)**^ **= 24.90** ***p*** **< .001** **φ = 0.48**	**χ**^**2(1)**^ **= 12.30** ***p*** **< .001** **φ = 0.33**	**χ**^**2(1)**^ **= 17.05** ***p*** **< .001** **φ = 0.39**
Groin	**χ**^**2(1)**^ **= 9.21** ***p*** **< .01** **φ = 0.29**	**χ**^**2(1)**^ **= 5.63** ***p*** **= .02** **φ = 0.23**	**χ**^**2(1)**^ **= 5.67** ***p*** **= .02** **φ = 0.23**	**χ**^**2(1)**^ **= 5.08** ***p*** **= .02** **φ = 0.22**
Lower leg and foot	χ^2^ [1] = 1.76 *p* = .18 φ = 0.13	**χ**^**2(1)**^ **= 22.85** ***p*** **< .001** **φ = 0.46**	**χ**^**2(1)**^ **= 15.12** ***p*** **< .001** **φ = 0.37**	**χ**^**2(1)**^ **= 18.95** ***p*** **< .001** **φ = 0.42**
Knee	χ^2^ [1] = 3.20 *p* = .07 φ = 0.17	**χ**^**2(1)**^ **= 36.46** ***p*** **< .001** **φ = 0.58**	**χ**^**2(1)**^ **= 20.30** ***p*** **< .001** **φ = 0.43**	**χ**^**2(1)**^ **= 31.02** ***p*** **< .001** **φ = 0.53**
Hip joint	**χ**^**2(1)**^ **= 23.00** ***p*** **< .001** **φ = 0.46**	**χ**^**2(1)**^ **= 10.54** ***p*** **< .01** **φ = 0.31**	**χ**^**2(1)**^ **= 13.82** ***p*** **< .001** **φ = 0.35**	**χ**^**2(1)**^ **= 12.26** ***p*** **< .001** **φ = 0.33**

Pearson’s chi-squared test (χ^2^) and 2-sided *p*-value together with mean square contingency coefficient (phi coefficient, φ). Significant differences are highlighted in bold (2-sided *p*-value < .05)

This finding is almost irrespective of the examiner.

Concerning the composition of the group for physical examination skills courses, students preferred self-assembled (males 62%; females 73%) over mixed gender (males 38%; females 21%) and same gender groups (males 5%; females 9% with multiple answers allowed).

In the free text answer section students could state their expectations and their fears. They pointed out that small groups, a short revision of the most important theoretical knowledge at the beginning, a well-prepared tutor, and structured feedback from the tutor provide the basis for a good examination skills course. Being examined by another person made them aware of how a patient feels. They recognise the benefit of learning these skills in a “safe” environment before being in contact with real patients. Nevertheless, the intimidating, and for some students embarrassing act of taking one’s clothes off in front of fellow students seems to have negative connotations.

## Discussion

This study elucidates the influence of not only assumable confounders such as gender and weight but also the role of religiousness in peer examination. Limitations of the study are its study design without information on longitudinal changes in the attitude of students towards peer examination and a small subgroup size of strongly religious students. Furthermore, as this study is strictly exploratory, a correction for multiple testing was not performed.

Intimate body regions are the breasts of females, genitals (including the pelvic organs and inguinal region) and the anus [15]. We also identified the breast and the groin as the most “socially sensitive” body parts, examination of which is unacceptable for a relevant number of students. Our questionnaire did not include the anus or the genitals, as these body regions are not part of the regular physical examination skills course, neither at our nor other German medical institutions. Rees et al. asked for the willingness to examine or be examined by a same gender or opposite gender peer on 12 different body regions (including genitals) [16]. In comparison, we also found that males are more willing than females to be peer examined. We can confirm, that the critical body regions for same gender peer examination comprise the breast, groin and hip, and additionally the upper body and the abdomen in the case of an opposite gender examiner. Our results also confirm the high level of student acceptance of > 95% for the head and neck, hand, arm and shoulder as well as knee examinations in the case of same gender peer examination as reported in previous studies [17, 18]. A religious background with strong beliefs still seems to lead to a lower willingness to be examined [19]. Students stated that this has to be respected but, despite this fact, everybody should take part in the examination courses. As do Rees et al., we see that religiousness plays an important role [17, 18]. In our study, religiousness was an important factor which influenced a student’s willingness to actively participate in the examination skills course. Strictly religious students were less willing to be examined even on otherwise uncritical body regions. Furthermore, this finding is independent of the examiner’s gender and professional status. Additionally, it should be remembered that in some cultures examinations of other parts of the body, e.g. eye, ear, nose and throat, might also be considered as intimate.

Surprisingly, BMI had no relevant effect on the willingness to be examined. Although self-reported BMI tends to be biased [20], we think that for the purposes of this study, two clearly divergent groups were investigated.

In the past, medical students learned examination of intimate body regions by examining patients under general anaesthesia, sometimes without the patients’ knowledge or consent [21]. Many physicians performed surgery under the assumption that patients had actually given or implied their consent to such examinations by coming to a teaching hospital. Others believe that patients will not consent if asked, making unauthorized training procedures necessary [22]. In contrast, many patients say that they would be willing to undergo pelvic examinations by medical students if asked [23, 24]. In the UK, the U.S. and Canada 37–46% of medical students learned to perform pelvic exams using unconscious patients awaiting surgery [25]. The guidelines of the Joint Commission on Accreditation of Health Care Organizations state that “participation by patients in clinical training programs should be voluntary” [26]. The Association of American Medical Colleges (AAMC) issued a press release in 2003 that called the use of women under anaesthesia without their knowledge and approval “unethical and unacceptable” [27].

A solution must be found to close this gap in the training of future physicians. Indeed, some people volunteer to become “professional patients”, placing their body at the disposal of medical students so that they can learn how to examine intimate parts [28]. So-called gynaecology teaching associates are used in the United States, Canada, Australia, and Scandinavia [25, 29, 30]. Hendrickx et al. conclude that working with intimate examination assistants represents a benefit in medical education by lowering the student’s threshold to perform physical examination on both genders [28].

Peer physical examination is a method which enables medical students to improve their skills by using each other as models. It helps them to become familiar with techniques before they see real patients in clinical settings [31]. However, 25% have difficulties and set limits with classmates [31]. Some of our students stated that they feared inappropriate behaviour by their classmates. They felt exposed when undressed as an examination model in front of a group of peers [31]. Abraham et al. point out that clear, direct communication about expectations regarding behaviour and the concepts of professionalism may help to improve classmate behaviour [32]. Direct attention to this issue in the tutorial setting might improve peer-group communication and minimise discomfort. To overcome the issue of being examined by a fellow student, we asked about the willingness to be examined by a student or by a professional tutor in front of the group. However, this did not lead to a higher acceptance. On the contrary, we found a general tendency towards less acceptance of professional tutors. Students are less willing to be examined by a tutor in front of the class than by a peer in a smaller group. We therefore recommend that this technique should not be performed in front of the whole class, but rather in small groups. In the free text area students wrote that they prefer learning new skills in a “safe” environment before being in contact with real patients. Wearn et al. state that peer examination helps to specify skills, allowing students more time to persist or repeat the examination until they “get it right”, reducing the potential harm caused to patients [11]. Generally, it has to be taken in account, that learning what is “normal” by examining healthy students is helpful before examining patients with abnormal findings [19]. Our students expect well-prepared tutors, who should repeat some theoretical knowledge at the beginning, demonstrate the examination and serve as supervisor.

Overall, we have shown, similarly to other studies, that students value the opportunity to learn basic skills by examining fellow students. Almost all (98%) agreed that peer physical examinations are appropriate, valuable, and a comfortable experience [31]. There was a high rating for the importance and motivation to take part. Our students focused on the benefits of learning these skills in a “safe” environment before being in contact with real patients. As time goes by, students’ opinions and attitudes have changed, and statements like those found by O′ Neill et al. were not found in our group. Students in O’Neill’s study cited the free availability of patients as a reason against students learning on one another and expressed the view that examination of normal people was artificial and irrelevant to students’ learning needs [19].

Further studies are desirable, investigating larger study populations to elucidate the role of possible confounders in detail, e.g. by use of multivariate analysis. Furthermore, it should be investigated whether particularly strongly religious students were likewise less willing to examine other students as being examined themselves.

## Conclusions

While motivation and acceptance of the physical examination skills course appears to be high, willingness to participate in peer physical examination is rather low for specific regions of the body. Critical regions are especially the breast and groin, followed by the upper body and hip. While gender certainly has an impact, the degree of religiousness in particular seems to influence a student’s propensity to volunteer for examination. Most students would prefer to choose their team for physical examination courses themselves rather than be assigned to a group, even if it is a same gender group. This study identified critical body regions for which self-selected groups or simulated patients might be an option. Students point out that small groups, a short revision of the most important theoretical knowledge at the beginning, a well-prepared tutor and structured feedback from the tutor are the basis for a good clinical skills course.
